# Comparative Analysis of Chilling Injury in Banana Fruit During Storage: Physicochemical and Microstructural Changes, and Early Optical-Based Nondestructive Identification

**DOI:** 10.3390/foods14081319

**Published:** 2025-04-11

**Authors:** Hui Ma, Lingmeng Hu, Jingyuan Zhao, Jie He, Anqi Wen, Daizhu Lv, Zhi Xu, Weijie Lan, Leiqing Pan

**Affiliations:** 1 College of Food Science and Technology, Nanjing Agricultural University, Nanjing 210095, China; 2022208019@stu.njau.edu.cn (H.M.); 2022808083@stu.njau.edu.cn (L.H.); 2023208020@stu.njau.edu.cn (J.Z.); 2023108028@stu.njau.edu.cn (J.H.); 2024808124@stu.njau.edu.cn (A.W.); 2 Analysis and Testing Center, Chinese Academy of Tropical Agricultural Sciences, Haikou 571101, China; ldz162000@126.com (D.L.); honic@yeah.net (Z.X.); 3 Sanya Institute of Nanjing Agricultural University, Sanya 572024, China

**Keywords:** chilling injury, banana fruit, quality property, optical technology, nondestructive identification

## Abstract

Chilling injury (CI) during postharvest storage seriously impairs bananas’ quality and marketability. This study systematically investigated CI mechanisms through physicochemical, microstructural, and optical analyses and innovatively developed a hyperspectral imaging (HSI)-based approach for early CI detection. Bananas stored at suboptimal (7 °C) and optimal (13 °C) conditions exhibited distinct physicochemical changes. CI progression was related to increased browning symptoms, an abnormal moisture redistribution (reduced pulp moisture content), and delayed softening. Microstructural analysis revealed membrane destabilization, cellular lysis, intercellular cavity formation, and inhibited starch hydrolysis under chilling stress. Hyperspectral microscope imaging (HMI) captured chilling-induced spectral variations (400–1000 nm), enabling the t-SNE-based clustering of CI-affected tissues. Machine learning models using first derivative (1-st)-processed spectra achieved a high accuracy. Both PLS-DA and RF had a 99% calibration accuracy and 98.5% prediction accuracy for CI classification. Notably, HSI detected spectral signatures of early CI (2 days post-chilling treatment) before visible symptoms, achieving a 100% identification accuracy with an optimized PLS-DA combined with 1-st processing. This study provides a theoretical basis for studying fruit CI mechanisms and a novel nondestructive optical method for early CI monitoring in postharvest supply chains.

## 1. Introduction

Banana (*Musa* spp.), a prominent tropical fruit, is renowned globally for its nutritional richness, appealing taste, and substantial economic importance [1,2]. However, the postharvest management of banana fruits remains challenging, particularly in regions where cold storage is essential for extending their shelf life. Chilling injury (CI), a physiological disorder, occurs when banana fruits are exposed to temperatures below their optimal range (typically < 13 °C). This exposure leads to a decline in fruit quality and a reduction in market value [3,4]. Cold stress induces varying degrees of quality degradation in both the peel and pulp of bananas, manifesting as surface pitting, internal browning, firmness loss, and aroma loss [5]. The cellular membrane, a critical barrier that maintains a stable intracellular environment for biochemical processes [6], is disrupted during CI, leading to physiological and metabolic disruptions. Previous studies have identified changes in sugar metabolism, acid metabolism, and membrane lipid composition, which led to the accumulation of harmful metabolites and membrane peroxidation [3,7]. Despite progress in metabolic and genomic studies on CI mechanisms, the initial microscopic changes associated with CI are not easily observable on the fruit’s surface, making timely intervention difficult. Therefore, a more comprehensive understanding of CI development, from the macroscopic to the microscopic scales, is necessary.

In recent years, optical technologies have demonstrated great potential for rapidly detecting fruit quality at both macroscopic and microscopic scales [8]. These technologies provide nondestructive, real-time monitoring capabilities, capturing subtle changes in fruit properties and serving as invaluable tools for studying CI mechanisms. CI adversely effects fruit’s physicochemical and quality parameters, altering the cellular microenvironment and changing light absorption and scattering patterns [9]. This provides a basis for applying optical methods in fruit quality detection. Spectral optical parameters offer valuable insights into physicochemical and structural variations in fruits. Specifically, the absorption coefficient (*μa*) and reduced scattering coefficient (*μs’*) are widely used to describe the energy loss per unit distance in fruit tissues [10]. These parameters have been successfully applied in pear disease identification [11], peach maturity classification [12], and nectarine CI detection [13]. Numerous quality prediction models based on optical properties have been established, enriching the application of optical technologies in nondestructive fruit detection. For example, visible–near-infrared (Vis–NIR) spectroscopy has been used to identify banana fruits infected with *Colletotrichum musae* [14]; NIR spectroscopy has been applied to distinguish banana ripening methods [15]; and Raman spectroscopy has been used for selective starch detection in bananas [16]. Additionally, the effective application of hyperspectral microscope imaging (HMI) technology in fast and accurate apple quality assessment [17], and the utility of hyperspectral imaging (HSI) combined with artificial neural networks in peach CI detection [18], further demonstrate the ability of optical technologies to capture key information related to fruit quality and structural changes. Unlike traditional spectroscopy techniques (Raman/NIR), which provide point-based measurements averaged over a larger area, HMI combines the spatial resolution of microscopy with the spectral resolution of HSI, enabling the acquisition of detailed spectral information at a microscopic scale. This allows for the detection of localized changes in fruit tissues, such as cellular-level alterations, which are critical for understanding the progression of CI. In contrast, Raman spectroscopy provides detailed molecular information through vibrational modes but the features are weak, while NIR spectroscopy is effective for bulk chemical analysis but cannot resolve microstructural changes. Although HSI provides spatial and spectral information, it operates at a macroscopic level, whereas HMI focuses on microscopic features, offering unique advantages for studying subcellular changes. Consequently, various optical technologies have distinct advantages in quality detection at macroscopic/microscopic and spatial/point scales. Nevertheless, research on the optical detection of CI in bananas using spectroscopic methods is limited. Therefore, investigating optical changes at both microscopic and macroscopic scales in response to CI using optical technologies is essential for understanding the compositional and structural evolution during CI.

The primary objective of this study is to investigate the mechanisms underlying CI in bananas and establish an early CI identification method. A multifaceted approach combining physicochemical, microscopic, and optical techniques has been used to elucidate macroscopic to microscopic changes in bananas during CI. Specifically, physicochemical properties, including the moisture content of peel and pulp, the weight loss rate of the whole fruit, peel color, and pulp firmness, were comprehensively investigated, along with changes in cell wall microstructural integrity and optical properties. A discriminant model based on HMI has been developed to reveal the correlation between CI development and the optical response of banana tissue. Additionally, the early nondestructive identification of CI in bananas using HIS combined with chemometrics highlights the significant potential of optical technologies for rapid and accurate fruit quality monitoring.

## 2. Materials and Methods

### 2.1. Materials

Banana (*Musa* spp.) samples were procured from a local wholesale market. Commercially mature bananas (70−80% maturity) with a uniform size, intact peel, and green-yellow coloration (~CIE L* 60, a* −13, b* 40) were selected, excluding specimens with mechanical damage or visible pathogens. Bananas were divided into individual banana fingers. Samples were randomly allocated into two groups. (i) Chilling treatment (CH) group: stored at suboptimal temperature (7 °C, 85% RH). (ii) Control (CTR) group: stored at optimal temperature (13 °C, 85% RH). For each treatment group (CH and CTR), the samples were stored for 0, 2, 4, 6, 8, and 10 days, respectively. Samples were stored in temperature- and humidity-controlled chambers (CTHI-250B, STIK Instrument, Shanghai, China). Prior to analysis, the samples were equilibrated at approximately 25 °C for 6 h to simulate retail display conditions.

### 2.2. Physicochemical Property Analysis

#### 2.2.1. CI Index

The CI index was evaluated based on the size of the browning area on the surface of banana fruit, according to a previous study with some modifications [3]. Each test sample contained 12 fruits. The CI index consists of the following five grades: 0 = no CI; 1 = 0 < browning area ≤ 25%; 2 = 25% < browning area ≤ 50%; 3 = 50% < browning area ≤ 75%; and 4 = browning area > 75%. The CI index was calculated by reference to the following formula: CI index = ∑(CI grade × CI number)/(4 × total number).

#### 2.2.2. Weight Loss Rate

The weight loss rate of whole fruits was measured by electronic balance (PMK223ZH/E, OHAUS, Changzhou, China) weighing. Specially, the mass of 5 fresh bananas was recorded as the initial mass. The calculation formula for the weight loss rate was as follows: weight loss rate (%) = (*W*0 − *W*)/*W*0 × 100, where *W*0 represents the initial mass, and *W* represents the sampling mass.

#### 2.2.3. Peel Color

The L*, a*, and b* values of banana peel were determined by a portable digital colorimeter (CR-10, Minolta, Tokyo, Japan) following the operating instructions. Measurements were taken at three points spaced approximately 120° apart around the equatorial part of the banana, and the average value was calculated as the final measurement.

#### 2.2.4. Moisture Content

Moisture content was determined separately for peel and pulp tissues using the oven drying method [17]. Briefly, approximately 0.5 g of banana sample (peel or pulp) was weighed (recorded as *M*0) and dried in a drying oven (DGG-9123A, SENXIN, Shanghai, China) at 100 °C until a constant weight (recorded as *M*1) was achieved. The moisture content was then calculated using the following formula: moisture content (%) = (*M*0 − *M*1)/*M*0 × 100, where *M*0 represents the initial mass and *M*1 represents the dried mass. For each treatment at every storage interval, 10 biological replicates (*n* = 10) were analyzed.

#### 2.2.5. Pulp Firmness

Pulp firmness was assessed using a texture analyzer (TMS-Pro, FTC, Sterling, VA, USA). Specifically, the peel of the banana was removed, and the pulp was placed horizontally on the substrate of the texture analyzer. A 6 mm diameter cylindrical probe was employed. The starting force was set at 0.1 N, the moving speed was set at 1 mm/s, and the pressing depth was set at 10 mm. Three equidistant measurement points along the longitudinal axis (proximal, medial, distal regions) were analyzed. Peak force (N) values were averaged as the determined firmness value. For each treatment at every storage interval, 8 biological replicates (*n* = 8) were analyzed.

### 2.3. Microstructural Property Analysis

Banana samples (peel or pulp) with a length and width of approximately 3 mm × 2 mm and a thickness of about 2 mm were cut using a sharp double-edged blade. The obtained samples were placed in a 2.5% glutaraldehyde fixative solution and fixed at 4 °C for 24 h. The dehydration treatment of the samples was carried out using ethanol solutions with different concentration gradients (30%, 50%, 70%, 90%, 95%, and 100%), with each concentration gradient for 20 min. After drying in a vacuum freeze-dryer (Alpha 2-4LSC Plus, Martin Christ, Osterode am Harz, Germany) for 48 h, the samples were mounted onto the scanning stage. The samples were observed using a scanning electron microscope (SU8010, Hitachi, Tokyo, Japan).

### 2.4. Optical Property Analysis

#### 2.4.1. Hyperspectral Microscope Imaging

A hyperspectral microscope imaging (HMI) system (Figure S1), which was constructed by integrating a hyperspectral imaging (HSI) system (with a spectral resolution of 5 nm in a range of 381.68–1040.41 nm and a halogen light source; LAMBDA-VN-EDU, Wuxi Spectrum Vision, Wuxi, China) with an upright microscope (MX4R, Wuxi Spectrum Vision, Wuxi, China), was utilized to acquire the spectral information of the microscopic structure of the pulp cells. Thin slices of banana pulp tissues (with a total of 89 bananas (14–15 samples per storage timepoint) for the CTR group and 87 bananas (14–15 samples per storage timepoint) for the CH group, respectively; each fruit measured in triplicate), approximately 0.2 mm in thickness, were prepared using a sharp double-edged blade. These slices were placed on glass slides and covered with cover slips. Subsequently, they were placed on the stage of a microscope. The cell morphology was observed under a 20-fold objective lens. The microscope was connected to an HSI system via a computer to obtain the microscopic spectra of the cell surfaces. The acquisition mode for the HMI of the pulp was the reflection mode. When the HMI system was in operation, the following parameter settings were applied: the exposure time was set to 25 ms, and the gain was set to 3%. To reduce or eliminate the influence of useless information in the original spectral data, such as noise, background color, uneven sample surface, baseline drift, and absorption peak overlap [19,20], and further improve the accuracy and stability of subsequent analysis and model establishment, 1st derivative (1-st) and 2nd derivative (2-nd) pretreatment methods were used to improve the spectral analysis accuracy. Subsequently, according to a ratio of calibration set/prediction set = 3:2, the samples were divided, and the discriminative models of cold damage and healthy samples based on SVM, PLS-DA, and RF were established for the spectral data.

#### 2.4.2. Hyperspectral Imaging

Hyperspectral images were acquired using a Vis–NIR HSI system, which consisted of a CCD camera (ICLB1620, Imperx, Boca Raton, FL, USA) with 804 × 440 pixels, an imaging spectrometer (ImSpector V10E, Specim, Oulu, Finland) with a spectral resolution of 1.3 nm in the 382.67–1010.64 nm range, a halogen light source (3900 ER, Illumination Technologies Inc., Syracuse, NY, USA), a mobile platform (IRCP0076 of-ICOMB001, Isuzu, Taiwan, China), and image acquisition software (Spectral Image-V10E, Isuzu, Taiwan, China). In the process of hyperspectral image acquisition, the light source was first turned on and preheated for 30 min to ensure that the temperature and light intensity were consistent during the acquisition process. The light source and camera lens were 30.0 cm and 24.0 cm away from the sample, respectively. The two hyperspectral light sources were fixed at 45 ° above the sample slope, which effectively reduced the shadow area. The light source intensity was set to 90 W. In order to avoid image distortion and ensure a sufficient light intensity on the sample surface, the exposure time was set to 3 ms, and the moving speed of the platform was set to 6.2 mm/s. The images of the positive and negative sides of each banana sample (with a total of 84 bananas (14 samples per storage timepoint); each fruit measured in triplicate) were collected by line sweep mode, and the average value of the extracted spectrum was calculated. The entire banana was defined as the region of interest (ROI) for the extraction of the average spectrum. The average spectrum of all the pixels in each ROI was regarded as the original spectrum of that region. The total number of pixels and the valid spectral values were counted, and the average spectrum was extracted for subsequent data processing and analysis. In addition, to eliminate unnecessary information and noise in the original spectrum, the multiplicative scatter correction (MSC), standard normal variate (SNV), and 1-st pretreatment methods were used to improve the accuracy and reliability of the spectral data. Subsequently, according to a ratio of calibration set/prediction set = 3:2, the samples were divided, and the discriminative models of CI samples based on SVM, PLS-DA, and RF were established for the spectral data.

### 2.5. Statistical Analysis and Software

All physicochemical property analyses were performed in triplicate (*n* = 3) at each storage time for both temperatures (7 and 13 °C), unless otherwise stated, and the data are expressed as the mean ± standard deviation (SD). Least significant differences (L.S.D.s) were calculated to compare significant effects at *p* < 0.05 using SPSS (version 22.0, SPSS Inc., Chicago, IL, USA).

## 3. Results and Discussion

### 3.1. Changes in Physicochemical Properties

**Figure 1:**
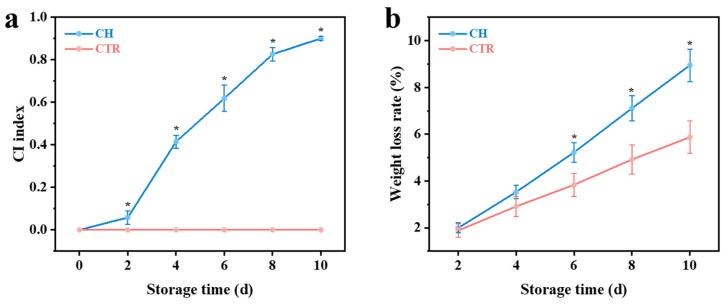
Changes in the (**a**) CI index and (**b**) weight loss rate of bananas under CTR and CH conditions during storage. “*” indicates significant differences (*p* < 0.05) between CTR and CH samples. CH, chilling treatment at 7 °C; CTR, control at 13 °C.

**Figure 2:**
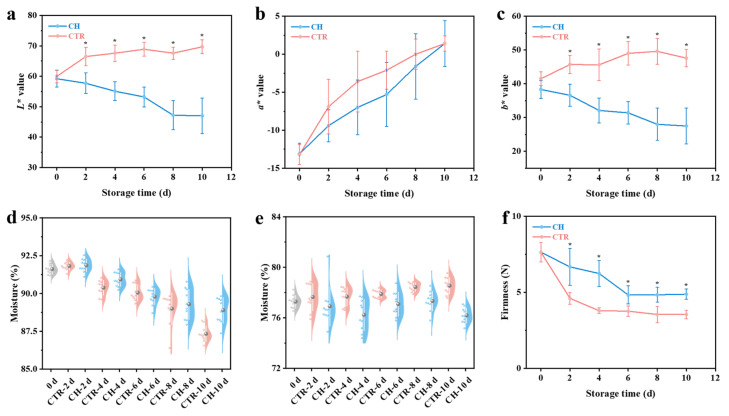
Changes in the (**a**–**c**) CIELAB space color coordinates (L*, lightness; a*, red–green; and b*, yellow–blue) and (**d**) moisture content of banana peel; and (**e**) moisture content and (**f**) firmness of banana pulp during storage. “*” indicates significant differences (*p* < 0.05) between CTR and CH samples. CH, chilling treatment at 7 °C; CTR, control at 13 °C.

Figure 1 and Figure 2 present a detailed comparison of the physicochemical properties of banana fruits stored under CTR and CH conditions. Throughout the storage period, the CTR group did not display any symptoms of CI. Conversely, bananas in the CH group showed a progressive increase in the CI index (Figure 1a; the fruit appearance is shown in Figure S2), indicating the onset and intensification of CI, which is consistent with previous observations [3]. This increase was accompanied by a higher weight loss rate in the CH group compared to the CTR group (Figure 1b). The elevated weight loss in the CH group is likely attributed to accelerated moisture evaporation under chilling stress, which disrupts cellular integrity and metabolic activity [3,21].

Color parameters (L*, a*, and b*) in the CIELAB space were used to evaluate the ripening and CI-related changes in banana peel. The a* value (red–green) increased in both groups during storage, indicating a gradual loss of green color as the fruit ripened (Figure 2b). However, the L* (lightness) and b* (yellow–blue) values exhibited contrasting trends between the CTR and CH groups (Figure 2a,c). For CTR samples, L* and b* values increased, reflecting the normal ripening process and development of a bright yellow color. In contrast, the CH group showed a decline in L* and b* values, indicating reduced peel glossiness and the onset of browning (Figure S2), which are typical symptoms of CI [3,22,23]. These findings align with previous studies reporting that chilling stress inhibits normal ripening and promotes peel browning [3,21].

The moisture content of both the peel and pulp was significantly affected by storage conditions. In the peel, the moisture content gradually decreased over time in both groups, with the CTR group showing a more substantial reduction (4.25%) compared to the CH group (2.72%) (Figure 2d). This difference can be explained by the normal ripening process in the CTR samples, where enhanced metabolic activity and water evaporation occur in the later stages of storage [23]. In contrast, chilling stress likely suppressed these metabolic processes, leading to a slower decline in peel moisture content. In the pulp, however, an opposite trend was observed (Figure 2e). The moisture content of the CTR group increased slightly during storage, possibly due to water migration from the peel to the pulp as the fruit ripened. Conversely, the CH group exhibited a decrease in pulp moisture content, suggesting that chilling stress disrupted the normal water distribution and absorption processes within the fruit.

Firmness, a critical indicator of fruit softening and senescence [24], was significantly influenced by storage conditions. Both groups exhibited a gradual decrease in firmness over time, but the CH group showed a slower rate of softening compared to the CTR group (Figure 2f). This suggests that chilling stress delayed the ripening process, which is consistent with the observed suppression of metabolic activity [3,21]. Although this delay in softening may seem beneficial for extending storage life initially, the associated CI and abnormal physiological metabolism could ultimately compromise the fruit quality and lead to uneven ripening or textural defects [22].

These results demonstrate that chilling stress during storage induces significant physicochemical alterations in banana fruits, as evidenced by progressive increases in CI index, weight loss, peel browning, abnormal moisture redistribution, and delayed softening kinetics. These findings reveal the dual nature of chilling stress impacts, where suboptimal temperatures simultaneously accelerate quality deterioration while delaying normal ripening processes, underscoring the critical importance of monitoring the occurrence of CI.

### 3.2. Changes in Microstructural Properties

**Figure 3:**
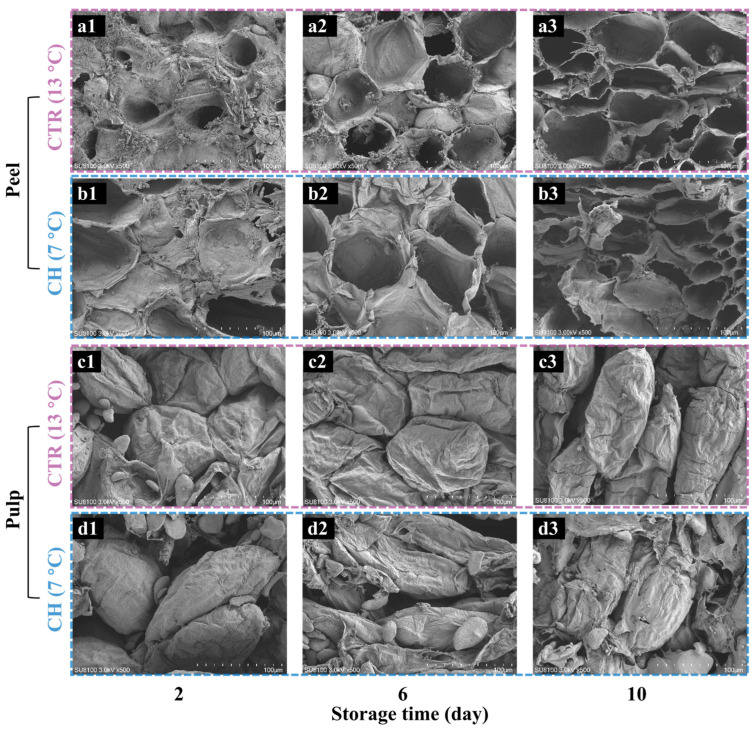
Changes in the microstructure of bananas under CTR and CH conditions during storage. SEM images of (**a1**–**a3**,**b1**–**b3**) peel and (**c1**–**c3**,**d1**–**d3**) pulp. (**a1**–**a3**,**c1**–**c3**) and (**b1**–**b3**,**d1**–**d3**) represent CTR and CH samples, respectively. (**a1**,**b1**,**c1**,**d1**), (**a2**,**b2**,**c2**,**d2**), and (**a3**,**b3**,**c3**,**d3**) represent samples stored for 2, 6, and 10 days. CH, chilling treatment at 7 °C; CTR, control at 13 °C.

Figure 3 systematically illustrates the temporal evolution of microstructural alterations in banana peel and pulp tissues under CTR and CH storage conditions, captured at three critical timepoints (2, 6, and 10 days). Scanning electron microscopy (SEM) images revealed significant differences in cellular integrity between the two groups, providing critical insights into the mechanisms underlying CI. In the CTR group, banana peel cells maintained their structural integrity during the initial storage phase (2 days), with intact plasma membranes and a closely interlocked cell arrangement pattern (Figure 3(a1)). Conversely, the peel of the CH group exhibited localized plasma membrane damage and an irregular cell arrangement (Figure 3(b1)), indicating the early stage of CI [9]. By day 6, although both groups exhibited membrane degradation (Figure 3(a2,b2)), the CH samples showed accelerated structural disorganization, featuring a fragmented membrane and collapsed epidermal layers. At the end of storage (day 10), the CTR peel cells still retained partial membrane integrity (Figure 3(a3)), while the CH peel showed extensive membrane rupture and cytoplasmic leakage (Figure 3(b3)). These observations suggest that prolonged chilling stress destabilized the cell structure of the peel tissue, leading to an irreversible loss of membrane functionality and accelerated cellular senescence. In the pulp tissue, the CTR samples exhibited progressive starch hydrolysis (Figure 3(c1–c3)), mediated by *α*-amylase activity during normal ripening [25]. By day 10, the CTR pulp showed complete starch conversion (Figure 3(c3)), which correlated with the accumulation of soluble sugar [26]. In contrast, the CH pulp maintained abundant starch granules (Figure 3(d1–d3)), indicating significant *α*-amylase inhibition at 7 °C [25]. This inhibition of starch–sugar metabolism corresponds to reduced softening rates (Figure 2f) and indicates the arrest of starch–sugar metabolism under chilling stress. Additionally, the CH pulp developed extensive cellular lysis and intercellular cavities (Figure 3(d3)), attributable to chilling-induced water redistribution and membrane damage [9]. These microstructural alterations imply potential correlations with physicochemical parameters: (i) a cell morphology change may affect the abnormal moisture distribution and membrane rupture strongly associated with the development of a browning peel [9]; (ii) delayed starch hydrolysis could delay tissue softening [23].

These findings establish a comprehensive structure–function relationship, bridging macroscopic quality parameters with microscopic structures under CI progression in banana fruit. Thus, subtle changes in the microstructure and physicochemical parameters of fruit tissue suggest the possibility of interpreting CI by optical techniques [10,17].

### 3.3. Identification of Chilling Injury Using Hyperspectral Microscope Imaging

**Figure 4:**
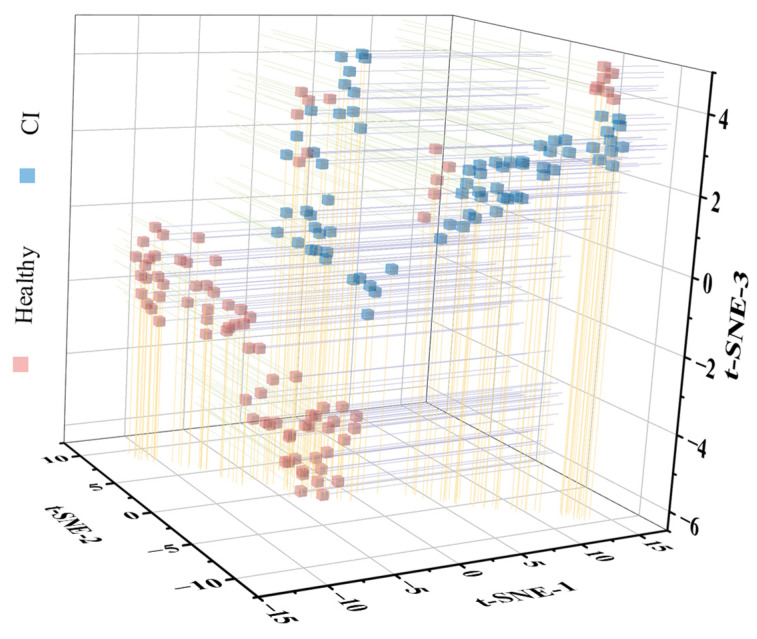
T-SNE analysis of banana pulp cells based on HMI. CI bananas include those in the CH group that were stored for 2, 4, 6, 8, and 10 days, and healthy bananas include those in the CH group that were stored for 0 days and the CTR group. CI, chilling injury; CH, chilling treatment at 7 °C; CTR, control at 13 °C.

During storage, CI triggers substantial physicochemical and microstructural alterations in banana fruits, leading to measurable changes in optical transmission properties. These changes include the degradation of chlorophyll (peel turns green and yellow), starch–sugar metabolism, and cellular disorganization, collectively modifying photon transmission patterns [11,27]. HMI technology was employed to quantify chilling-induced alterations in the optical reflectance properties of pulp cells. Figure S3a presents the complete set of raw reflectance spectra from banana pulp samples (including both healthy and CI samples), demonstrating characteristic variations across the 400–1000 nm spectral range. Figure 4 demonstrates the t-distributed stochastic neighbor embedding (t-SNE) dimensionality reduction of high-dimensional HMI data, effectively visualizing spectral patterns in three-dimensional space for an intuitive cluster analysis [28]. The t-SNE visualization revealed distinct spectral clusters, with a clear separation between chilling-injured and normal cells in the reduced dimensional space. This robust clustering demonstrates the sensitivity of HMI to subtle compositional and structural changes induced by CI. Similar phenomena have been observed in previous studies, which found that the 800–1000 nm spectral range is capable of capturing variations in cell wall structures, and the spectral intensity shows strong correlations with parameters such as moisture content (at 982 nm) and L* values (at 946 nm) [17]. These results collectively highlight the intrinsic relationship between spectral changes and alterations in fruit cell structure and quality parameters.

Table 1 systematically compares the performance of machine learning models in classifying CI versus healthy fruits by using HMI-derived spectral features. Figure S3a presents the raw reflectance spectra. The variations in the NIR region (750–1000 nm) partially reflect cellular structure modifications and the moisture content redistribution [17,29], which accounts for the distinct t-SNE clustering patterns. To improve the extraction of spectral features, Savitzky–Golay derivatives (1-st and 2-nd) were applied. This approach effectively reduced baseline drift and random noise, while preserving critical spectral features [30]. Figure S3b and Figure S3c, respectively, present the preprocessed spectra after the 1-st and 2-nd transformations, highlighting the enhanced spectral features for subsequent analysis. Three machine learning models, namely SVM, PLS-DA, and RF, were developed using raw, 1-st, and 2-nd preprocessed spectra to evaluate the effectiveness of HMI in CI detection. As shown in Table 1, the models based on raw spectra achieved a calibration accuracy of 89.2–93.8% and a prediction accuracy of 82.3–87.7%, among which RF showed the best performance (calibration: 93.8%; prediction: 87.7%). The derivative preprocessing significantly enhanced the performance of the models. PLS-DA and RF, combined with the 1-st, achieved a calibration accuracy of 99% and a prediction accuracy of 98.5%, which can be attributed to effective baseline correction and noise reduction [30]. These results demonstrate that HMI is capable of capturing the variations in photon transmission associated with quality and structural changes induced by CI. These findings lay a foundation for the optical-based rapid detection of CI in banana fruits.

### 3.4. Early Nondestructive Identification of Chilling-Injured Banana Fruit Using Hyperspectral Imaging

**Figure 5:**
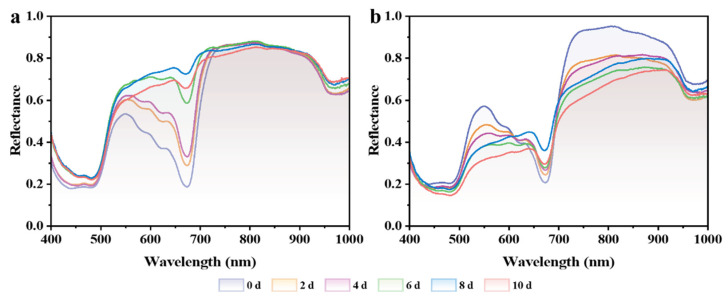
Average reflectance spectra of bananas under (**a**) CTR and (**b**) CH conditions during storage measured by HSI. CTR, control at 13 °C; CH, chilling treatment at 7 °C.

The early detection of CI in bananas is crucial for implementing timely storage interventions, potentially reducing postharvest losses. A nondestructive approach based on HSI technology was developed for early CI detection in banana fruits. Figure 5 presents the average reflectance spectra (400–1000 nm) of bananas under CH and CTR conditions during 0–10 days of storage, revealing distinct spectral patterns. In the CTR group (Figure 5a), the initial low reflectance at 673 nm (the chlorophyll absorption peak) gradually increased during storage. This was accompanied by enhanced reflectance in the ~550 nm (yellow–orange) region, indicating chlorophyll degradation and color change from green to yellow [31,32]. In the CH group (Figure 5b), reduced reflectance in the 500–600 nm range indicated the development of browning, while the limited changes at 673 nm suggested inhibited chlorophyll degradation [31]. These observations are consistent with membrane damage and abnormal ripening [3]. Notably, significant spectral differences are detectable as early as 2 days after the CH was applied. This demonstrates the capability of HSI to capture early CI-related changes before a visible quality deterioration occurs. Therefore, early detection at 2 days after the CH exposure is critical for timely intervention, potentially preventing the subsequent quality deterioration.

**Figure 6:**
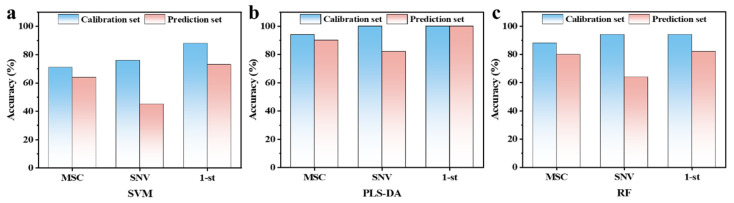
Early (2d) nondestructive identification accuracy of CI bananas using HSI combined with (**a**) SVM, (**b**) PLS-DA, and (**c**) RF models. MSC, multiplicative scatter correction; SNV, standard normal variate; 1-st, 1st derivative; CI, chilling injury.

Initial analysis using HSI-based PLS-DA models with 1-st preprocessing achieved a 100% calibration accuracy and 92.6% prediction accuracy (Figure S4). This remarkable performance demonstrated the excellent CI detection capability of the models during the 0–10-day storage period. These results are comparable to those of a previous study, which reported a satisfactory accuracy (95.8% for both training and testing sets) for predicting CI in peaches using HSI (400–1000 nm) combined with a multilayer perceptron artificial neural network (MLPANN) [18]. Additionally, the detection of CI in cucumber has been successfully achieved through a dual-band radio algorithm and a PCA model within a narrow spectral region of 733–848 nm [33]. Previous studies based on HSI (750–850 nm) have also demonstrated successful CI identification in avocado [34]. Therefore, these findings underscore the effectiveness of integrating HSI (400–1000 nm) with chemometrics for precise and robust CI identification. However, the early detection of CI remains challenging due to the subtle visible symptoms in fruits during the initial stages. To address this, CI progression stage (0/2/4/6/8/10 days; the occurrence time of CI) classification models, including MSC, PLS-DA, and RF, were developed, with detailed performance metrics presented in Table S1. The PLS-DA model with 1-st preprocessing demonstrated superior performance, achieving a 99.0% calibration accuracy and 98.5% prediction accuracy in CI stage classification. The potential of HSI technology (400–1000 nm) in classifying CI severity has also been confirmed in cucumbers, with an overall accuracy of 91.6% [35]. These results firmly establish HSI as a powerful tool for CI stage identification throughout the storage period. Figure 6 presents the early CI detection (2 days) results using HSI technology, highlighting the model performance across different preprocessing methods. Both the PLS-DA and RF models combined with MSC preprocessing outperformed the SVM model, showing a higher accuracy in both calibration and prediction. SNV preprocessing resulted in overfitting, with the calibration accuracy significantly exceeding the prediction accuracy across all models. The 1-st preprocessing significantly improved its performance, effectively reducing spectral noise and enhancing feature extraction [19,20]. The improvement results of the 1-st preprocessing on the HSI spectral-based modeling method have also been observed in the CI severity identification of kimchi cabbage [36]. In this work, the PLS-DA model achieved optimal performance, with a 100% accuracy in both the calibration set and prediction set, demonstrating an exceptional early CI detection capability. These results demonstrate that PLS-DA combined with 1-st preprocessing provides a reliable method for the early detection of CI in banana fruits. To our knowledge, this study is the first to propose a nondestructive approach for early CI detection in bananas using HSI (400–1000 nm) integrated with chemometrics. However, it should be noted that the spectral range of 874–1374 nm has been shown to capture critical information related to CI in kimchi cabbage [36], indicating the presence of multiple spectral features associated with CI across the full HSI range of 400–2500 nm. This suggests the potential for optimizing early CI prediction based on HSI by selecting feature wavelengths within the 400–2500 nm range. Furthermore, ANN models may outperform PLS-DA, as evidenced by the discrimination of CI in kimchi cabbage based on feature wavelengths, where the average accuracy of PLS-DA was <91.2%, compared to >92.4% for ANN [36]. Nevertheless, the current technology enables rapid, real-time, and nondestructive quality monitoring, facilitating timely adjustments to storage conditions and minimizing postharvest losses.

## 4. Conclusions

In this study, a comprehensive investigation of the CI mechanism of banana fruits was conducted by integrating physicochemical, microstructural, and optical analyses. Additionally, for the first time, a method for the early nondestructive discrimination of CI in bananas was proposed. The development of CI was characterized by increased browning symptoms, an abnormal moisture redistribution, and delayed softening, all of which collectively contribute to the quality deterioration. Microstructural examinations revealed critical degradation pathways, including membrane destabilization, cellular lysis, and intercellular cavity formation, establishing a direct link between microscopic damage and macroscopic quality loss. To address the aggravation of CI, optical-based identification methods were investigated. HMI successfully captured chilling-induced spectral variations (400–1000 nm), enabling a clustering based on t-SNE. The PLS-DA and RF models, which utilized 1-st-processed spectra, achieved exceptional accuracy, reaching a 99% calibration accuracy and 98.5% prediction accuracy for CI classification. Furthermore, HIS combined with PLS-DA was successfully applied to detect the spectral signatures of early-stage CI, achieving a 100% identification accuracy. These findings promote the postharvest management of tropical fruits by clarifying the mechanism of CI occurrence and proposing an immediate optical-based nondestructive identification method. Future work should focus on improving the prediction of even earlier stages of CI and enhancing model performance by selecting key wavelengths from the long-wave spectral range (1000–2500 nm) and combining them with advanced machine learning algorithms to achieve a higher recognition accuracy and faster data processing speeds.

## Figures and Tables

**Table 1:** Comparison of classification accuracy of SVM, PLS-DA, and RF models for healthy and CI bananas based on HMI. CI bananas include those in the CH group that were stored for 2, 4, 6, 8, and 10 days, and healthy bananas include those in the CH group that were stored for 0 days and the CTR group. Raw, raw spectra; 1-st, 1st derivative; 2-nd, 2nd derivative; CI, chilling injury; CH, chilling treatment at 7 °C; CTR, control at 13 °C.

Model	Preprocessing	Calibration Set Accuracy (%)			Prediction Set Accuracy (%)		
		Healthy	CI	Overall	Healthy	CI	Overall
SVM	Raw	91	87	89.2	79	86	82.3
	1-st	86	98	92	75	98	85.4
	2-nd	86	97	91.5	77	98	87.5
PLS-DA	Raw	94	93	93.8	87	80	83.3
	1-st	98	100	99	97	100	98.5
	2-nd	91	100	95.4	90	98	93.7
RF	Raw	98	89	93.8	91	83	87.7
	1-st	100	98	99	97	100	98.5
	2-nd	100	95	97.9	97	93	95.4

## Data Availability

The original contributions presented in this study are included in the article/Supplementary Materials. Further inquiries can be directed to the corresponding authors.

## Supplementary Materials

The following supporting information can be downloaded at: https://www.mdpi.com/article/10.3390/foods14081319/s1, Figure S1. Hyperspectral microscope imaging system. Figure S2. Changes in appearance of bananas under CH and CTR conditions during storage. CH, chilling treatment at 7 °C; CTR, control at 13 °C. Figure S3. HMI data. (a1) Raw spectra. (a2) Spectra with 1-st preprocessing. (a3) Spectra with 2-nd preprocessing. 1-st, 1st derivative; 2-nd, 2nd derivative. Figure S4. Classification accuracy of PLS-DA model for CI and healthy bananas using HSI based on 1-st preprocessed spectra. CI bananas include those in the CH group that were stored for 2, 4, 6, 8, and 10 days, and healthy bananas include those in the CH group that were stored for 0 days and the CTR group. 1-st, 1st derivative; CI, chilling injury; CH, chilling treatment at 7 °C; CTR, control at 13 °C. Table S1. Comparison of classification models for CI bananas with different storage days. MSC, multiplicative scatter correction; SNV, standard normal variate; 1-st, 1st derivative; CI, chilling injure.
