# Supplementary material for: Comparative Analysis of Chilling Injury in Banana Fruit During Storage: Physicochemical and Microstructural Changes, and Early Optical-Based Nondestructive Identification
Source: Foods. 2025 Apr 11;14(8):1319. doi: 10.3390/foods14081319 (PMC12026267; doi:10.3390/foods14081319)
Supplement: Supplementary file 1 [file foods-14-01319-s001.zip › foods-3538522-supplementary.pdf]

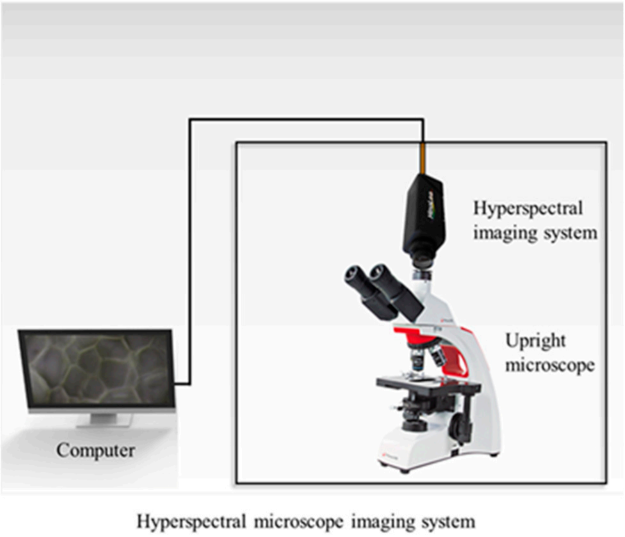

Figure S1. Hyperspectral microscope imaging system.

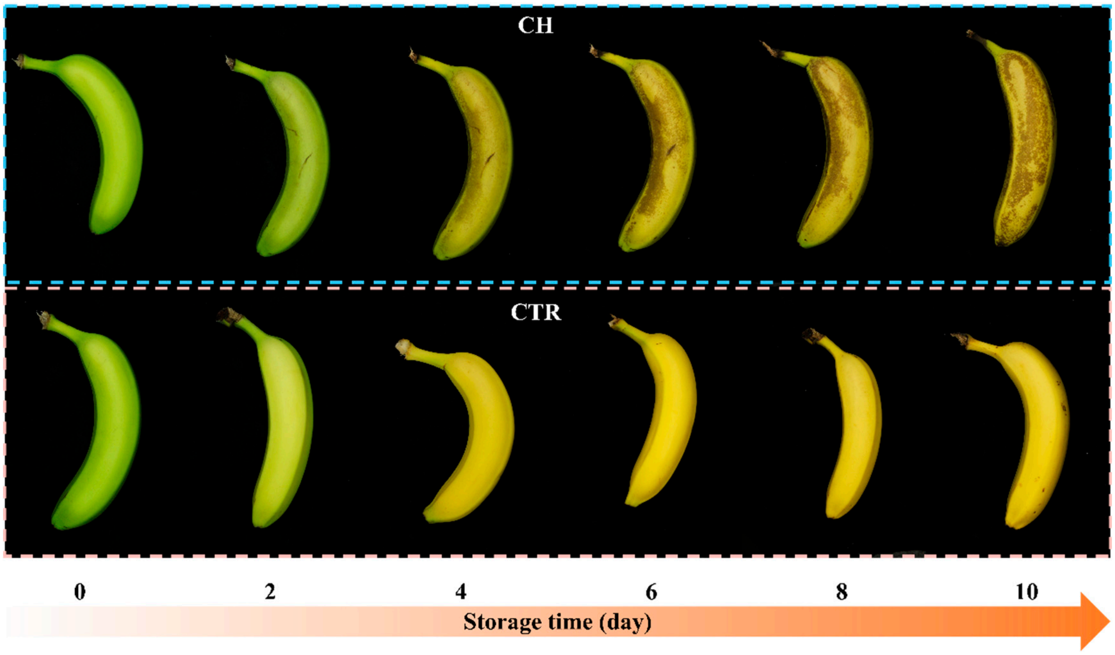

Figure S2. Changes in appearance of bananas under CH and CTR conditions during storage. CH, chilling treatment at 7 °C; CTR, control at 13 °C.

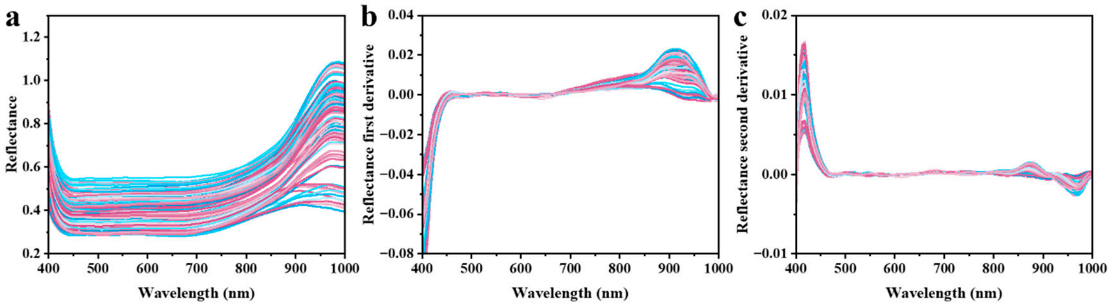

Figure S3. HMI data. (a1) Raw spectra. (a2) Spectra with 1-st preprocessing. (a3) Spectra with 2-nd preprocessing. 1-st, 1st derivative; 2-nd, 2nd derivative.

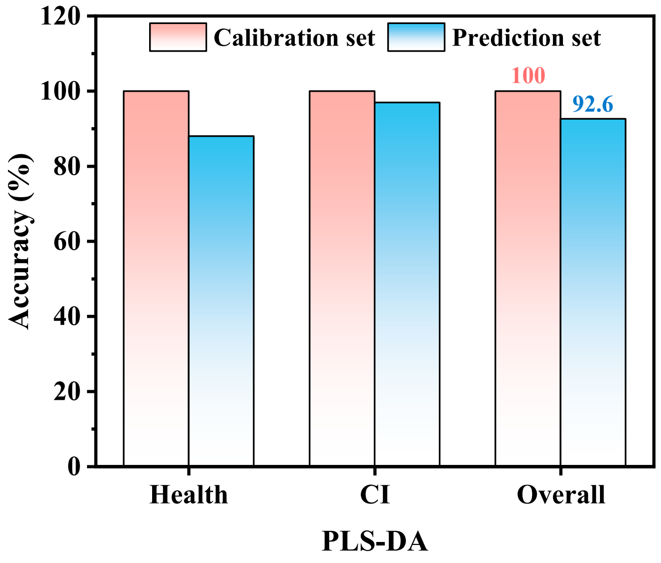

Figure S4. Classification accuracy of PLS-DA model for CI and healthy bananas using HSI based on 1-st preprocessed spectra. CI bananas include those in the CH group that were stored for 2, 4, 6, 8, and 10 days, and healthy bananas include those in the CH group that were stored for 0 days and the CTR group. 1-st, 1st derivative; CI, chilling injury; CH, chilling treatment at 7 °C; CTR, control at 13 °C.

Table S1. Comparison of classification models for CI bananas with different storage days. MSC, multiplicative scatter correction; SNV, standard normal variate; 1-st, 1st derivative; CI, chilling injure.

Model	Preprocessing	Calibration set accuracy (%)							Prediction set accuracy (%)						
		0 d	2 d	4 d	6 d	8 d	10 d	Overall	0 d	2 d	4 d	6 d	8 d	10 d	Overall
SVM	MSC	100	71	94	100	100	100	94.1	100	64	100	91	100	91	90.9
	SNV	100	76	94	88	100	100	93.1	100	45	100	91	100	91	87.9
	1-st	100	88	94	100	100	100	97.1	100	73	100	100	100	100	95.5
PLS-DA	MSC	100	94	88	100	94	100	96.1	100	90	91	100	89	98	94.7
	SNV	100	100	82	100	73	100	92.4	100	82	76	100	94	94	91.0
	1-st	**100**	**100**	**94**	**100**	**100**	**100**	**99.0**	**100**	**100**	**100**	**100**	**91**	**100**	**98.5**
RF	MSC	100	88	100	88	94	100	95.1	78	80	57	60	70	68	68.8
	SNV	100	94	100	94	94	100	97.1	100	64	82	82	73	55	75.8
	1-st	100	94	100	100	100	100	99.0	82	82	82	91	73	91	83.3
